# Immunometabolism in Tuberculosis

**DOI:** 10.3389/fimmu.2016.00150

**Published:** 2016-04-21

**Authors:** Lanbo Shi, Eliseo A. Eugenin, Selvakumar Subbian

**Affiliations:** ^1^Public Health Research Institute, New Jersey Medical School, Biomedical and Health Sciences, Rutgers – The State University of New Jersey, Newark, NJ, USA

**Keywords:** the Warburg effect, hypoxia-inducible factor 1 alpha, metabolism, tuberculosis, granuloma, immune response

## Abstract

Immunometabolism, the study of the relationship between bioenergetic pathways and specific functions of immune cells, has recently gained increasing appreciation. In response to infection, activation of the host innate and adaptive immune cells is accompanied by a switch in the bioenergetic pathway from oxidative phosphorylation to glycolysis, a metabolic remodeling known as the Warburg effect, which is required for the production of antimicrobial and pro-inflammatory effector molecules. In this review, we summarize the current understanding of the Warburg effect and discuss its association with the expression of host immune responses in tuberculosis (TB), an infectious disease caused by *Mycobacterium tuberculosis* (*Mtb*). We also discuss potential mechanisms underlying the Warburg effect with a focus on the expression and regulation of hypoxia-inducible factor 1 alpha (HIF-1α), the regulatory subunit of HIF-1, a major transcription regulator involved in cellular stress adaptation processes, including energy metabolism and antimicrobial responses. We also propose a novel hypothesis that *Mtb* perturbs the Warburg effect of immune cells to facilitate its survival and persistence in the host. A better understanding of the dynamics of metabolic states of immune cells and their specific functions during TB pathogenesis can lead to the development of immunotherapies capable of promoting *Mtb* clearance and reducing *Mtb* persistence and the emergence of drug resistant strains.

## Tuberculosis

Tuberculosis (TB), caused by the intracellular pathogen *Mycobacterium tuberculosis* (*Mtb*), has become the top killer disease alongside HIV/AIDS, responsible for 1.5 million deaths in 2014 (1). In the infected host, the outcome following *Mtb* infection is determined by both host- and pathogen-derived factors and by their interactions (2, 3). One of the pathological hallmarks of TB is the formation of the granuloma, an orderly aggregation of host immune cells around the infected macrophage(s), because of complex host–pathogen interactions at the site of infection (4, 5). A key factor for the success of *Mtb* as a pathogen is its ability to survive and persist in host cells within the granulomas for long periods of time and to exacerbate pathological progression, which ultimately results in bacillary spread within and between the hosts (4). As a focus of the disease, mycobacterial granulomas have been the subject of intense studies mainly aimed at understanding the mechanisms of their formation, function, maintenance, and evolution. Gaining a better understanding of these processes will not only shed light on the host and pathogen factors involved in TB pathogenesis but also facilitate the development of novel pathogen- and/or host-directed therapeutic strategies to eliminate TB.

## Granuloma Formation and Maintenance

Tuberculoma or granuloma during *Mtb* infection is formed by an orchestrated series of events involving host chemokines and cytokines that coordinate the recruitment of immune cells from circulation and their accumulation at the infection foci. During initial stages of the granuloma formation, chemokines and cytokines produced mainly by the infected alveolar macrophages and dendritic cells (DCs) bring about a focal recruitment and accumulation of mononuclear cells (4, 6). Although fully activated phagocytes are capable of killing *Mtb*, the pathogen can survive and proliferate in the phagocytes by inhibiting their innate immune functions (7–9), for instance, by interfering with the phagosome–lysosome maturation and acidification pathways (10) and by inducing immune suppressive functions of the infected host cells, e.g., by elevating IL-10 production (11). Modulation of innate immune cell functions by *Mtb* can also lead to altered T cell-mediated immune functions (9, 12), which are an important factor responsible for the delayed initiation and activation of adaptive immunity during *Mtb* infection in the lungs (13–15).

With the accumulation of activated T lymphocytes, the granuloma becomes a fully organized structure, containing a central area of *Mtb*-infected macrophages surrounded by freshly recruited, non-infected phagocytes and lymphocytes. In the vicinity of this highly cellular “solid” granuloma, macrophages are activated toward a M1 phenotype by the cytokines and antimicrobial effector molecules produced by the cells of innate and adaptive immunity, which ultimately curtails intracellular bacterial growth and drives the infection into latent/persistent stage (5, 16, 17). When the infection progresses toward active disease, *Mtb* alters the macrophage polarization toward a M2 phenotype, which is associated with anti-inflammatory properties and elevated lipid metabolism that contributes to the formation of foamy macrophages (18). This process facilitates necrosis of immune cells at the center of the granuloma, enhancing the possibility of *Mtb* dissemination. Although granulomas have been traditionally regarded as cellular structures beneficial to the host that seal off the infection and focus the immune response to a limited area, recent reports indicate that tuberculous granulomas also act as survival niches for *Mtb*, and that the host–pathogen interactions in the granulomas play key role in the expansion and dissemination of infection (4, 19). Thus, formation, maintenance, and evolution of granulomas are now regarded as a balance between antimicrobial effectors of host immune cells and the ability of *Mtb* to withstand these bactericidal factors and cause disease.

The central role of host–pathogen interactions in TB progression is supported by multiple reports, including recent findings that the ESAT-6/CFP-10 complex, a major virulence factor of pathogenic *Mtb*, plays critical roles in driving the granuloma evolution in a zebrafish infection model (20). This notion is also supported by the diminished and/or altered pathophysiology caused by some persistent phenotype mutants of *Mtb* (such as *sigE* and *sigH* deletion mutants), which are associated with decreased recruitment of T cells or adaptive immunity but are independent of *in vivo* bacterial growth (21, 22). Modulation of granuloma formation and maintenance by *Mtb* is further underscored by the differential outcome of infection by two clinical *Mtb* strains of different virulence (23, 24). In the rabbit model of TB that mimics several aspects of human TB, including the formation of well-differentiated granulomas, ranging from necrotic, caseating, and cavitating to healing lesions, pulmonary infection by a hypervirulent *Mtb* strain HN878 results in active disease in the lungs of rabbit, marked with high bacillary load and destructive disease pathology (24). In contrast, infection by a hyper-immunogenic *Mtb* CDC1551 strain cannot sustain the high bacterial numbers after protracted initial growth, and the infection establishes a latent stage with time, characterized by undetectable level of bacillary load and absence of lung pathology; however, these latently infected animals can reactivate bacillary growth and disease pathology upon immune suppression treatment (23, 25). Understanding various cellular and molecular components of granuloma formation, development, and evolution and their roles in protecting the host will thus be of paramount significance for the development of preventive and therapeutic strategies against TB.

## The Warburg Effect and Immune Cell Functions

Recently, there has been an increasing appreciation of the importance of the relationship between the bioenergetic pathways and immune cell functions. Thus, immunometabolism has emerged as a new field of investigation focusing on understanding how and why immune cells commit to a particular metabolic fate to support or direct functional changes. Unlike other cells, immune cells are required to stay in a relatively quiescent state under normal physiological conditions and to activate and mount rapid response and effector functions upon infection or under pathological conditions. Recent reports indicate that differential metabolic signatures are essential for specific effector functions of cells of both innate and adaptive immune systems (26, 27). For example, as an essential component of innate immunity, the macrophage undergoes reprogramming to two predominant functional phenotypes: the classically activated (M1) and the alternatively activated (M2) states (28, 29). The M1 macrophages activated in response to interferon-γ (IFN-γ) and toll-like receptor (TLR) ligands, such as lipopolysaccharide (LPS), generate pro-inflammatory cytokines that potentiate the activation and differentiation of inflammatory, Th1 type adaptive immune cells (28). Metabolically, M1 cells display enhanced glycolysis and decreased oxygen consumption (30, 31). A comprehensive metabolic map of LPS-activated macrophages shows upregulation of glycolytic genes and downregulation of mitochondrial genes, which correlates directly with the profiles of altered metabolites (32). Perturbation of glycolysis, for instance, by blocking the monocarboxylate transporter 4 (MCT4) (a major lactate secretion transporter) in LPS-activated macrophages, diminishes nitric oxide (NO) production and the expression of pro-inflammatory cytokines (33). In contrast, the M2 macrophages formed in response to Th2 cytokines, such as IL-4 and IL-13, are characterized by higher levels of scavenger receptors and anti-inflammatory cytokines (34), and these cells’ metabolic profiles are similar to those of non-polarized, resting macrophages, in which mitochondrial fatty acid oxidation and oxidative metabolism are the major carbon and energy sources (30). Similarly, metabolic reprogramming of DCs is also associated with their activation and functions (35). In immunogenic DCs, a metabolic switch characterized by increased glycolysis and concurrent decrease in oxidative phosphorylation in response to TLR stimulation was reported to be essential for their effective maturation and functions (36–39). However, in tolerogenic DCs, increased expression of genes involved in the mitochondrial oxidative phosphorylation (40, 41) is consistent with their functional phenotype marked with maturation resistance and increased level of immunoregulatory factors (42, 43), which are important for regulatory T cell response. Likewise, upon activation, effector T cells also reprogram their metabolism from an oxidative metabolism to a highly glycolytic and glutamine-dependent metabolic program, and this metabolic transition enables cell growth, proliferation, differentiation, and secretion of effector molecules, whereas oxidative phosphorylation is the dominant energy source in naive and regulatory T cells (44–47). Fatty acid oxidation was shown to be crucial for the development and survival of CD8^+^ memory and CD4^+^ regulatory T cells (47–50).

Enhanced glycolysis is generally accompanied with a concurrent increase of the pentose phosphate pathway (PPP) that provides the NADPH and ribose phosphate in activated immune cells (32, 36). This metabolic switch is reminiscent of the Warburg effect in cancer cells that predominantly utilize aerobic glycolysis instead of oxidative phosphorylation in mitochondria as the main route of ATP generation and recycling of NADH to NAD^+^ with the formation of lactate (51). Glycolysis not only produces ATP faster, albeit less efficiently, than oxidative phosphorylation but also provides biosynthetic precursors needed for rapid cell growth, proliferation, and cellular biosynthetic capacity (52). Thus, a shift to the Warburg effect supports a rapid and vigorous response during immune cell activation, such as activation of inflammatory response to infection, which involves not only rapid cell growth and proliferation but also the generation of pro-inflammatory cytokines and antimicrobial molecules, including reactive oxygen and nitrogen species (ROS and RNS).

The Warburg effect is regulated by the master transcription factor – hypoxia-inducible factor 1 (HIF-1) (53, 54). HIF-1 functions as a heterodimer that comprised a highly regulated HIF-1α and constitutively expressed HIF-1β subunits (55). Identified for its role in hypoxia, HIF-1 also plays a regulatory role in response to a variety of molecular signals of infection and inflammation even under normoxic conditions (56, 57). HIF-1α is induced by pro-inflammatory cytokines, growth factors, and a broad range of infections (57–62), and its induction is a general component of the host response to infection (63). For example, HIF-1α is required for the pro-inflammatory Th17 cell differentiation (64), the activation and regulation of glycolytic capacity in myeloid cells (65), and for the release of pro-inflammatory cytokines, expression of co-stimulatory molecules, and induction of the Warburg effect enzymes in DCs (66).

Hypoxia-inducible factor 1 alpha expression is regulated at both transcriptional and posttranslational levels. Members of the nuclear factor-κB (NF-κB) family constitute a major signaling pathway closely associated with *Hif-1a*/*HIF-1A* expression (57, 63). Posttranslational regulation is mainly mediated through the stabilization of HIF-1α protein. Under normal physiological conditions, the HIF-1α level is kept low by proteasome-mediated degradation after hydroxylation by three oxygen-dependent prolyl hydrolases (PHDs) (67). A factor inhibiting HIF (FIH), an aspariginyl hydrolase, also inhibits the transactivation function of HIF-1α (68). The PHDs belong to an α-ketoglutarate (2-oxoglutarate)-dependent dioxygenase superfamily that uses O_2_ as a co-substrate to add a hydroxyl group to specific proline residues within the HIF-α oxygen-dependent domains (67). Upon infection or during inflammation, increased *Hif-1a*/*HIF-1A* expression and inhibition of PHDs and/or FIH activity lead to elevated levels of HIF-1α, which in turn positively regulates several cellular processes, including myeloid cell infiltration and activation and the induction of glycolytic isoenzymes and glucose transporters (65, 66). Tricarboxylic acid (TCA) cycle intermediates also contribute to the stabilization of HIF-1α by inhibiting the PHDs (69). In particular, increased levels of succinate from the TCA cycle were shown to serve as an inflammatory signal to induce IL-1β by the stabilization of HIF-1α through the direct inhibition of PHDs in LPS-treated macrophages (32). HIF-1α stabilization was also shown to depend on ROS (70).

## The Warburg Effect in TB

### The Warburg Effect in Murine Models of TB

**Figure 1:**
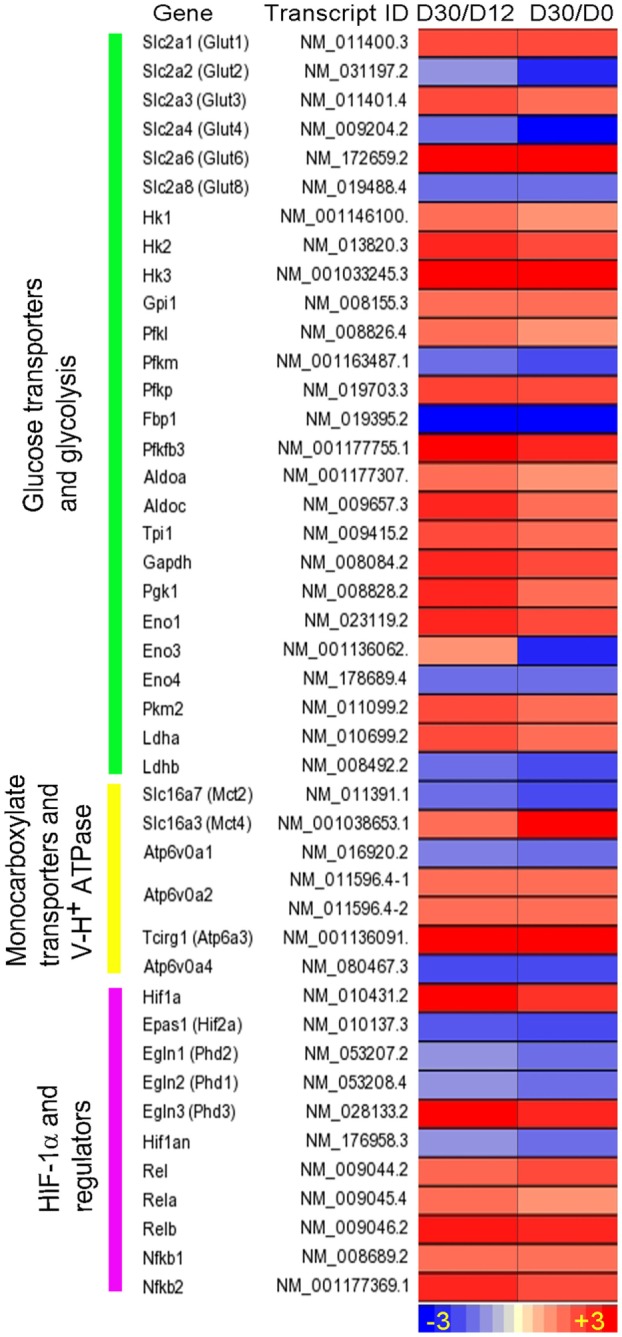
**Intensity plot of transcripts encoding enzymes/proteins involved in the Warburg effect and its regulation by HIF-1α-associated mechanism in *Mtb*-infected mouse lungs**. Shown are fold change in gene expression, derived from ratios of the normalized mRNA counts [fragments per kilobase of exon per million (FPKM) fragments mapped] at day 30 (D30) postinfection vs. D12 and at D30 vs. D0. WT C57BL/6 mice were infected with ~100 CFU of *Mtb* H37Rv *via* the respiratory route, and lung samples were harvested at specific points for RNA-seq. Data are derived from Shi et al. (71). Intensity plot was constructed using Partek Genomics Suite (PGS) ver 6.5. The color scale ranges from +3 (up; red) to −3 (down; blue). The genes are arranged according to their hierarchical position in the respective pathways.

We recently reported RNA-seq- and immunohistochemistry-based evidence of the Warburg effect in immune cells during early stages of granuloma formation in a murine model of low-dose aerosol *Mtb* infection (71) (Figure 1). Specifically, we observed that in response to *Mtb* infection, host central metabolism was characterized by a coordinated upregulation of genes encoding enzymes/isozymes reminiscent of the Warburg effect in cancer cells (53). These include genes encoding facilitative glucose transporters 1, 3, and 6 (GLUT1, 3, and 6), glycolytic enzymes/isozymes including hexokinases (HK2 and 3), members of phosphofructokinase (PFK) family 1 and 2, glyceraldehyde-3-phosphate dehydrogenase (GAPDH), phosphoglycerate kinase 1 (PGK1), enolase 1 (ENO1), lactate dehydrogenase A (LDHA), and MCT4. Gene expression profiles were corroborated by increased protein expression of the representative Warburg effect enzymes and H^+^-ATPase in host immune cells, suggesting an enhanced glycolytic flux in infected mouse lungs (71). Furthermore, ^1^H NMR-based metabolomics profiling revealed increased accumulation of lactate, the product of glycolysis, in the lungs of *Mtb*-infected mice (72). Elevated glycolytic flux was also supported by our recent finding of concurrent downregulation of pyruvate oxidation and oxidative phosphorylation in the mitochondria (71). We also observed a simultaneous upregulation of PPP in mouse lungs, which is in agreement with the metabolic state of activated immune cells (27, 73). Our study also revealed that the Warburg effect is concurrent with increased levels of mRNA and protein of HIF-1α in macrophages and T cells, suggesting a possible role of HIF-1α in regulating the Warburg effect during *Mtb* infection (Figure 1).

Given that the Warburg effect in mouse lungs occurs in parallel with the expression of host adaptive immunity (71, 74), our data suggest that the Warburg effect may constitute a bioenergetic requirement to mount effective host antimicrobial and immune responses to *Mtb* infection (74). This notion is in line with the recent findings that stabilization of the host HIF-1α, both pharmacologically and genetically, at early stages of *M. marinum* infection in zebrafish, is associated with reduced bacterial burden in infected larvae, and that increasing the HIF-1α signaling enhances RNS levels in neutrophils (75). The importance of HIF-1α is also underscored by the observation that a mouse strain lacking HIF-1α displays impaired resistance against *M. avium* infection and premature emergence of granuloma necrosis (76).

To corroborate observations from infected mouse lungs, we analyzed the transcriptome data from murine bone marrow-derived macrophages (MBDMs) infected by either of the two clinical *Mtb* strains, CDC1551 or HN878 (77). Given that these two clinical strains induce differential immune responses at the initial stage of the MBDM infection (77), a difference in the metabolic state of infected macrophages by these two strains was expected. Indeed, while a similar set of genes encoding the Warburg effect enzymes/isoenzymes and HIF-1α-associated signaling was induced in MBDMs infected by both strains (Table 1), we also noted a divergence in the glucose metabolism and glycolytic flux in the infected macrophages. In particular, while the early (6 h postinfection) upregulation of glucose transporter gene *Glut6* was similar in MBDM infected by these two strains, a heightened induction of *Pfkfb3*, which encodes the 6-phosphofructo-2-kinase/fructose-2,6-biphosphatase 3 (PFKFB3) of PFK-2 family, was observed in CDC1551-infected MBDMs (Table 1), similar to the results obtained from *Mtb*-infected mouse lungs (71) (Figure 1). As PFKFB3 has the highest kinase/phosphatase activity ratio among the PFK-2 members and its product fructose-2,6-diphosphate (F-2,6-BP) plays a key regulatory role for potentiating the glycolysis flux by relieving the inhibition of PFK-1 (78), a heightened induction of *Pfkfb3* in CDC1551-infected MBDMs is expected to result in enhanced the glycolytic flux. Thus, the high glycolysis state in *Mtb* CDC1551-infected MBDMs may constitute the metabolic basis for the robust early pro-inflammatory responses in these cells. Consistently, genes encoding inflammatory and antimicrobial effector molecules such as *Il12b*, *Ccl8*, *Cxcl9*, and *Nos2* were highly upregulated in these macrophages (77). In contrast, a relatively low activation of the Warburg effect together with elevated glucose uptake observed in *Mtb* HN878-infected MBDMs may be associated with the induction of a dysregulated host cell lipid metabolism, which results in a less stressful intracellular environment for *Mtb* HN878, as described by Koo et al. (77).

**Table 1:** **Change in the transcripts encoding facilitative glucose transporters, glycolytic enzymes, monocarboxylate transporters (MCTs), subunits of H^+^ V-ATPase, HIF-1α, and factors associated with HIF-1α regulation in murine MBDMs**.

Gene ID	Symbol	CDC1551 (6 h)	HN878 (6 h)	CDC1551 (24 h)	HN878 (24 h)
**Facilitative glucose transporters and glycolysis**					
10507594	*Slc2a1 (Glut1)*	**4.33**	**4.15**	**1.83**	**2.50**
10547641	*Slc2a3 (Glut3)*	**−2.44**	**−2.06**	**1.35**	−1.08
10481164	*Slc2a6 (Glut6)*	**12.37**	**11.89**	**2.04**	**2.51**
10369541	*Hk1*	**1.93**	**1.41**	**1.51**	**1.67**
10545588	*Hk2*	**3.11**	**2.52**	**2.49**	**2.52**
10409376	*Hk3*	**2.10**	**1.69**	**2.91**	**2.75**
10562360	*Gpi1*	**−1.75**	**−1.39**	**1.09**	**1.15**
10370376	*Pfkl*	**2.18**	**2.28**	**1.47**	**2.12**
10426557	*Pfkm*	**−1.85**	**−1.65**	−1.06	−1.04
10407481	*Pfkp*	**2.36**	**1.68**	−1.05	**1.16**
10602385	*Pfkfb1*	**−1.22**	**−1.19**	1.09	1.06
10357535	*Pfkfb2*	**−3.17**	**−2.23**	−1.05	−1.09
10480035	*Pfkfb3*	**10.27**	**4.34**	**2.13**	**2.28**
10589329	*Pfkfb4*	**−3.03**	**−1.80**	−1.09	**−1.21**
10568050	*Aldoa*	**−1.14**	1.03	**1.35**	**1.43**
10379153	*Aldoc*	**−1.75**	**−2.04**	**−1.31**	**1.37**
10547830	*Tpi1*	**−1.28**	**−1.15**	**1.58**	**1.80**
10601390	*Pgk1*	**1.22**	**1.29**	**1.67**	**1.85**
10450923	*Pgk2*	1.16	1.09	**1.29**	1.23
10473240	*Eno1*	**−1.13**	**1.19**	**1.28**	**1.59**
10547807	*Eno2*	**2.20**	**3.77**	**3.75**	**3.96**
10377938	*Eno3*	**−1.19**	**−1.17**	1.02	−1.02
10493382	*Pklr*	**1.25**	**1.19**	1.07	1.13
10585932	*Pkm2*	−1.08	1.05	**1.54**	**1.64**
10553301	*Ldha*	1.06	1.03	**1.27**	**1.46**
10549097	*Ldhb*	**−1.34**	**−1.33**	−1.17	−1.17
**Monocarboxylate transporters and V–H^+^ ATPase**					
10495035	*Slc16a1 (Mct1)*	**1.49**	**1.28**	**−1.44**	1.01
10372988	*Slc16a7 (Mct2)*	**−4.07**	**−4.06**	**−1.73**	**−2.42**
10383502	*Slc16a3 (Mct4)*	**1.97**	**2.08**	**1.74**	**2.83**
10464529	*Tcirg1* (*Atp6v0a3)*	**1.54**	**1.61**	−1.01	**−1.17**
10381187	*Atp6v0a1*	**−2.09**	**−1.37**	**−1.78**	**−2.14**
10525804	*Atp6v0a2*	**1.61**	**1.50**	**1.56**	**1.22**
**Hypoxia-inducible factor and regulation**					
10396421	*Hif1a*	**1.84**	**1.94**	**−1.11**	**1.58**
10447317	*Epas1 (Hif2a)*	**−1.31**	**−1.19**	**−1.24**	−1.07
10560329	*Hif3a*	−1.01	−1.10	1.11	**1.13**
10463380	*Hif1an*	**−1.55**	**−1.44**	−1.08	**−1.20**
10582712	*Egln1 (Phd2)*	**−1.75**	**−1.19**	**−1.40**	−1.11
10561170	*Egln2 (Phd1)*	**1.49**	**1.26**	**1.70**	**1.36**
10400304	*Egln3 (Phd3)*	**1.19**	**1.21**	**1.35**	**2.04**
10384725	*Rel*	**3.04**	**3.22**	**−1.43**	**−1.22**
10460631	*Rela*	**1.37**	**1.56**	**−1.12**	**−1.18**
10560575	*Relb*	**1.90**	**2.16**	1.07	**1.13**
10502299	*Nfkb1*	**3.38**	**3.37**	**−1.25**	**−1.16**
10463599	*Nfkb2*	**2.98**	**3.34**	**1.34**	**1.49**
10577560	*Ikbkb*	**1.27**	**1.18**	**−1.10**	**−1.09**

*The annotated and differentially expressed genes (bolded) were identified based on changes in average expression levels with a significance of *P* ≤ 0.05 (bolded). The gene expression ratios at 6 and 24 h postinfection were compared to the values of uninfected and calculated as median-centered values (from three independent experiments), and the log2 expression ratio was converted to fold change. Data were derived from microarray data [Gene Expression Omnibus (GEO) repository accession number GSE31734; Koo et al. (77)]*.

### The Warburg Effect in the Rabbit Model of TB

**Figure 2:**
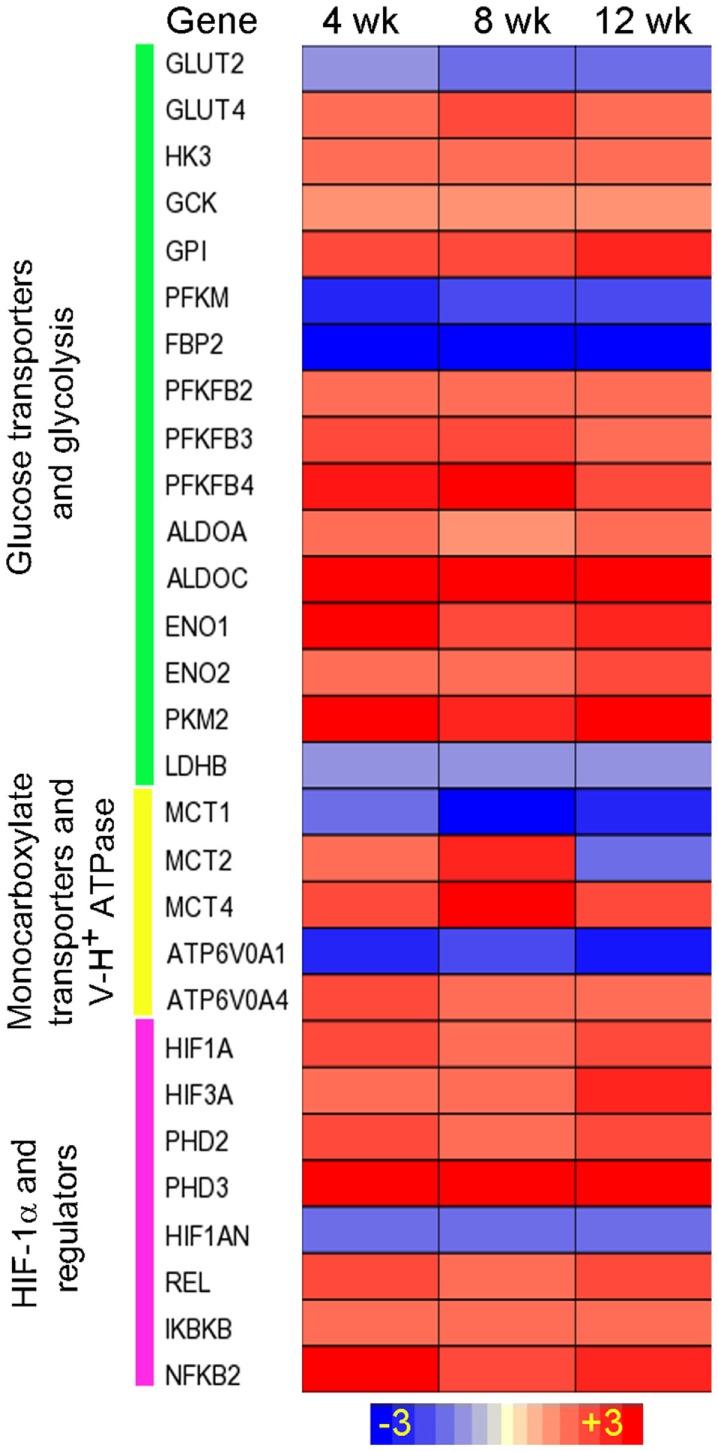
**Intensity plot of the Warburg effect-associated gene expression in *Mtb* HN878-infected rabbit lungs**. Microarray data from *Mtb* HN878-infected or uninfected rabbit lungs at 4, 8, and 12 weeks postinfection (*n* = 4 per time point per group) were normalized and analyzed using Partek Genomics Suite (PGS) ver 6.5. Data shown are fold change in gene expression in the infected, compared to uninfected rabbit lungs. Only significantly differentially expressed genes (*P* ≤ 0.05) are shown in the plot. Intensity plot was constructed using Partek Genomics Suite (PGS) ver 6.5. The color scale ranges from +3 (up; red) to −3 (down; blue). The genes are arranged according to their hierarchical position in respective pathways. Data are derived from GEO# GSE27992 and 33094 [Subbian et al. (24)].

We have established a rabbit model of pulmonary *Mtb* infection that closely mimics the pathological features of *Mtb* infection/disease seen in humans (24, 25, 79, 80). We analyzed the kinetics of lung transcriptome of rabbits infected aerogenically by *Mtb* HN878, in which lung pathology shows features of active disease with the development of necrotic granulomas and cavities (24). The transcriptome analysis clearly showed an upregulation of gene networks involved in inflammatory immune response and antimicrobial molecules production (24), concurrent with the upregulation of genes encoding the Warburg effect enzymes/isozymes between weeks 4 and 8 postinfection (Figure 2). *SLC2A4*, which encodes the facilitative glucose transporter member 4 (GLUT4), was induced in rabbit lungs. GLUT4 is implicated in insulin-regulated glucose uptake (81) and is associated with elevated glycolysis in cancers (82, 83). Moreover, three out of the four genes encoding the PFK-2 members, PFKFB2, PFKFB3, and PFKFB4, were induced in HN878 infected rabbit lungs (Figure 2). As both PFKFB2 and PFKFB4 have much lower 6-phosphofructo-2-kinase to fructose-2,6-bisphosphatase activity ratio than PFKFB3 (78, 84), the formation of F-2,6-BP by these two enzymes could be very limited (84); thus, their contribution to glycolysis is expected to be restricted. In addition, alongside the induction of MCT4, *SLC16A7* encoding the MCT2, was also induced in HN878-infected rabbit lungs. As MCT2 has much higher affinity for pyruvate than MCT4 (85), its induction could be related to the pyruvate metabolism in mitochondria. Indeed, knockdown of MCT2 was shown to result in mitochondrial dysfunction, cell-cycle arrest, and senescence in cancer cells (86). In the HN878-infected rabbit lungs, as the disease progresses with exacerbated disease pathology, sustained level of high bacillary load, and a compromised host immune response at 12 weeks postinfection and beyond (24), expression of the Warburg effect enzymes/isoenzymes and HIF-1α remained at elevated levels (Figure 2). These transcriptome data suggest that progression of active disease is also accompanied with an elevated Warburg effect in infected rabbit lungs, perhaps driven by the elevated bacillary load that can serve as potent antigen to stimulate macrophages and lymphocytes. More in-depth studies are needed to understand whether upregulation of different Warburg effect isozymes and MCTs contributes to the different immune responses elicited by the two *Mtb* strains in the infected rabbit lungs.

### The Warburg Effect in the Lung Granulomas of Patients with Active TB

To extend our observation of the Warburg effect in multiple models of TB and its possible association with the host immune response during *Mtb* infection, we analyzed the transcriptome data from the cavitary lung granulomas of human patients with active TB and compared them to the corresponding profiles from uninvolved portions of the same lungs (87). The genome-wide transcriptional profiling of the human lung TB granulomas revealed significant upregulation of network/pathway genes associated with immune cell movement, IL-17-mediated inflammatory response, and STAT1-mediated T cell activation (87). Moreover, genes involved in vitamin D receptor (VDR) signaling and interferon signaling were upregulated and enriched in granulomas with higher bacillary loads relative to uninvolved lung tissue (87).

**Figure 3:**
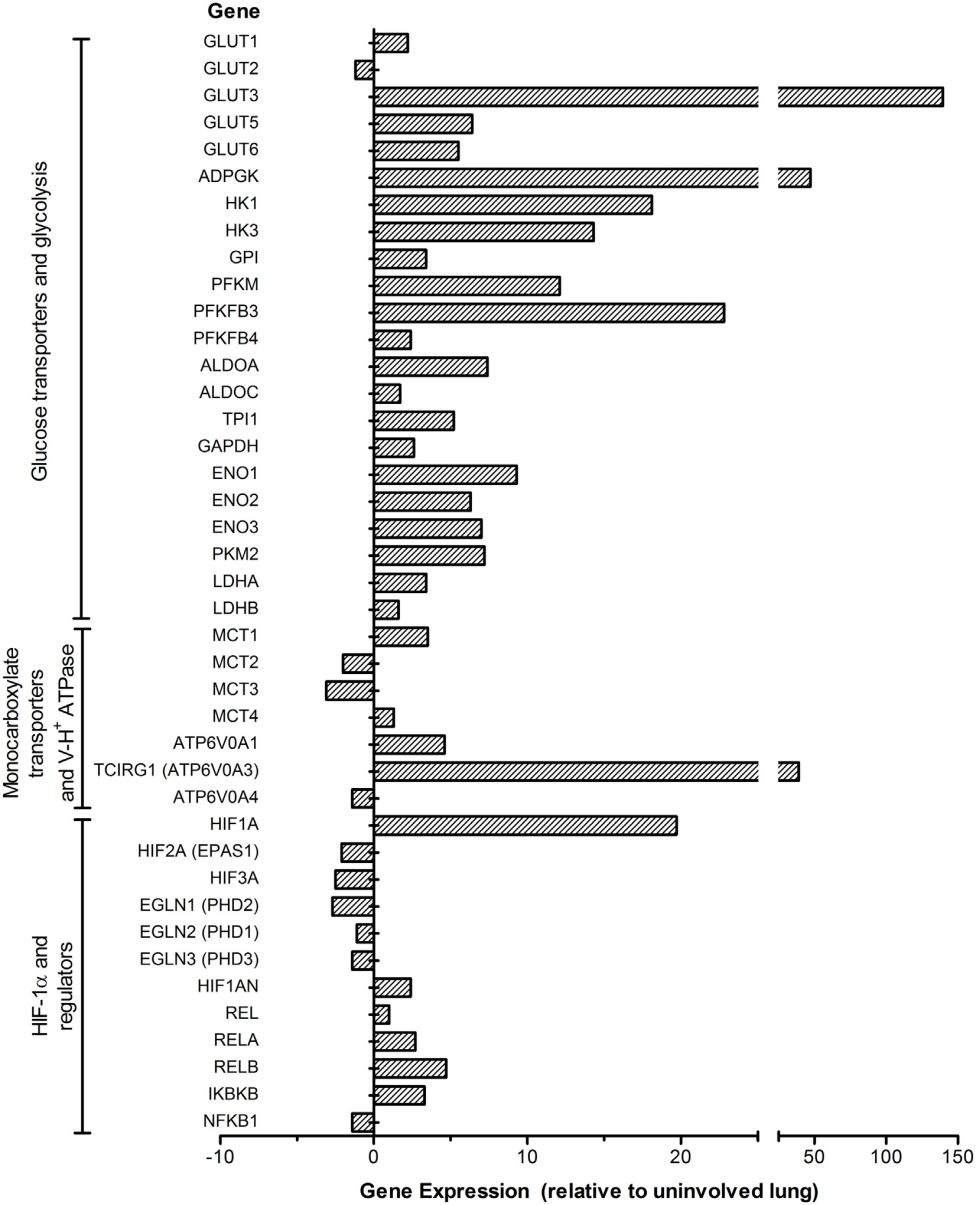
**Expression profile of the Warburg effect-associated genes in the lung granulomas of patients with active pulmonary TB**. Microarray data from granulomas or uninvolved lung areas were normalized, analyzed and plotted using Partek Genomics Suite (PGS) ver 6.5. Data shown are fold change in gene expression in the lung granulomas, compared to uninvolved lungs of pulmonary TB patients. Only significantly differentially expressed genes (*P* ≤ 0.05) are shown in the plot. The genes are arranged according to their hierarchical position in respective pathways. Data are derived from GEO# GSE20050 [Subbian et al. (87)].

Consistent with our findings in the model systems, a more inclusive set of genes encoding the enzymes/isozymes involved in the Warburg effect, HIF-1α regulation, and cytosolic pH regulation by H^+^-ATPase and MCTs were also found in the lung granulomas of patients with active TB (Figure 3). Specifically, multiple genes encoding GLUTs, including GLUT1, GLUT3, GLUT5, and GLUT6, were upregulated in active TB granulomas. *GLUT3* was induced to the highest degree among all glucose transporters, underscoring its potential role in glucose metabolism during active TB in human lungs. Among the glucose phosphorylation genes, alongside *HK1* and *HK3*, *ADPGK* encoding the ADP-dependent glucose kinase (ADPGK) was also highly upregulated. *ADPGK* is not a target of HIF-1 and does not appear to contribute to glycolysis in cell lines and cancer cells (88). However, T cell activation was shown to be dependent on ADPGK-driven enhanced glucose uptake and glycolysis and linked to mitochondrial ROS generation from the activation of respiratory-chain-associated glycerol-3-phosphate dehydrogenase 2 (GPD2) (89). Downregulation of ADPGK or GPD2 abundance was shown to inhibit oxidative signal generation and induction of NF-κB-dependent gene expression (89). Thus, ADPGK appears to be associated with the activation of T cells in the lung granulomas of patients with active TB.

Consistent with the observations in murine models, active TB in human lungs is also concurrent with a heightened induction of *PFKFB3*, encoding the key enzyme potentiating glycolytic flux through activating PFK-1 enzymes. The concurrent high induction of *PFKM* encoding PFK-M isoform of PFK-1 suggests its potential role in the glycolytic flux in the human lung granulomas. Moreover, alongside the induction of *MCT4*, *SLC16A1* ­encoding MCT1 was also upregulated in the granulomas of active TB patients, consistent with its ubiquitous expression in multiple types of primary cells and cell lines (90, 91). As MCT1 also functions in lactate uptake (90), its upregulation may also indicate increased lactate metabolism in the lung granulomas of active TB patients. In addition, the heightened induction of H^+^-ATPase involved in cytosolic pH homeostasis, as seen in mouse lungs (71), is also in agreement with increased expression of glycolysis genes in the lung granulomas of active TB patients (Figure 3). In accordance with the expression profile of the Warburg effect and immune activation network genes, immune cell profiling of these active TB granulomas showed higher numbers of CD3^+^/CD4^+^ T cells as well as CD68^+^ macrophages and multinucleated giant (MNG) cells relative to those in uninvolved lungs (87). Thus, our data strongly suggest a positive correlation between the Warburg effect and host immune response specifically in the granulomatous microenvironment, which is distinct from the uninvolved lung tissue adjacent to the granulomas. Understanding of how a change in the Warburg effect is associated with disease progression and/or therapeutic treatment can be harnessed to develop novel and effective therapeutic agents for TB treatment.

## Signaling Pathways and Host Factors Associated with the Warburg Effect during *Mtb* Infection

Multiple mechanisms have been reported to contribute to the increased HIF-1α expression and the Warburg effect during *Mtb* infection. NF-κB family proteins are the primary transcriptional regulatory factors involved in initiating/maintaining cellular response during host innate and adaptive immunity (92). These proteins regulate the transcription of various cytokine/chemokine genes as well as those involved in intracellular signaling, whose products are not only essential components of the immune response (92) but also crucial for the transcriptional activation of *Hif-1a*/*HIF-1A* (57, 63, 93). During *Mtb* infection, expression of the NF-κB family members was upregulated in macrophages, mouse and rabbit lungs, as well as in the granulomas of human pulmonary TB patients (71, 87) (Table 1; Figures 1–3). For the activation of NF-κB, IkappaB kinases (IKK), especially IKK-β, are required for the phosphorylation-induced degradation of NF-κB inhibitors in response to infection and inflammation, and IKK-β is also essential for HIF-1α accumulation in macrophages during bacterial infection (57). NF-κB proteins then dimerize and translocate into the nucleus to activate the transcription of *Hif-1α/HIF-1A* and other target genes (92). In agreement with these findings, we observed upregulation of *Ikbkb*/*IKBKB* during *Mtb* infection in murine MBDMs as well as in the lungs of mice and rabbits and in the lung granulomas of pulmonary active TB patients (24, 71, 87).

Another pathway of NF-κB signaling activation was shown to be associated with the induction of ROS from mitochondrial electron transport chain (mROS) due to the downregulation of oxidative phosphorylation (94). This process was associated with TLR-1-, -2-, and -4-mediated signaling that promotes and translocates the TNF-associated factor 6 (TRAF6) to mitochondria to potentiate mROS generation through evolutionarily conserved signaling intermediate in toll (ECSIT) pathways (94). The requirement for ECSIT and/or TRAF6 for mROS production was shown in *Salmonella typhimurium*-infected macrophages where a reduction in TLR-induced ROS impaired the ability of murine macrophages to kill the bacteria (94). Interestingly, *Mtb* infection also induced the expression of both *ECSIT* and *TRAF6* in murine macrophages and in the lungs of infected mice and rabbits as well as in the granulomas of human pulmonary TB patients (23, 24, 77, 87). However, the direct contribution of mROS to the antimicrobial immune response against *Mtb* and to the Warburg effect remains to be elucidated.

Several host factors contribute to the elevated expression of HIF-1α and the associated immune/metabolic responses during *Mtb* infection *in vitro* and *in vivo*. One such factor is NO, which is generated by the action of an inducible NO synthase (NOS2/iNOS) in infected murine macrophages and in the lungs of infected mice and rabbits (24, 77, 95). As a target of HIF-1α (60, 96), NOS2/iNOS induction may result in the redistribution of intracellular oxygen and inhibition of the PHDs, the negative regulators of HIF-1α (97), thus forming a positive feedback loop that leads to a sustained high level of HIF-1α and amplifies macrophage activation. Second, increased level of TCA cycle intermediate succinate in *Mtb*-infected mouse lungs (72) can elevate the level of HIF-1α by inhibiting the activity of PHDs (32). Third, decreased levels and/or functions of PHD1 and 2 due to the reduced transcription of these enzymes reported in *Mtb*-infected mouse lungs and active human TB lung granulomas (71, 87) (Figures 1 and 3) can contribute to the elevated levels of HIF-1α. Fourth, downregulation of *Hif-2a/HIF-2A*, encoding another oxygen-responsive component of the HIF family, can also indirectly enhance the level and/or function of HIF-1α during *Mtb* infection. Indeed, we have observed downregulation of *Hif-2a/HIF-2A* in *Mtb*-infected murine macrophages and mouse lungs as well as in the lung granulomas of active TB patients (71, 77, 87) (Figures 1 and 3; Table 1).

Hypoxia-inducible factor 2 alpha has physiologically antagonistic functions with HIF-1α (98), and its transcription is induced by Th2 cytokines and is associated with M2 macrophage polarization (98). Moreover, as HIF-2α also regulates the expression of ARG1, which competes with NOS2/iNOS for arginine, a common substrate for both enzymes (98), downregulation of HIF-2α indirectly favors M1 macrophage polarization by tipping the balance toward enhanced HIF-1α-mediated Warburg effect and the antimicrobial function against *Mtb*. Downregulation of HIF-2α can be related to the upregulation of *phd3/PHD3* during *Mtb* infection. In contrast to *phd1* and *phd2*, the expression of *phd3* was increased in *Mtb*-infected mouse lungs (71), consistent with the presence of a hypoxia-responsive enhancer element in *phd3* (99). In addition, expression of *phd3*/*PHD3* was also upregulated in *Mtb*-infected MBDMs and rabbit lungs (Table 1; Figure 2). Interestingly, because PHD3 best retains its function under prolonged hypoxia, compared to the other two paralogs, PHD1 and PHD2 (100), and exhibits a higher activity toward HIF-2α (101), its upregulation during *Mtb* infection can lead to specific degradation of HIF-2α. Thus, the divergent expression and regulation of HIF-1α and HIF-2α in response to *Mtb* infection suggests a synergy in activated host immune cells to maximize HIF-1α-mediated host antimicrobial and inflammatory response. Indeed, an inverse correlation between the bactericidal activity and the HIF-2α expression has been reported in activated neutrophils (75).

Enhanced HIF-1α expression and its roles in the regulation of the Warburg effect and antimicrobial response during *Mtb* infection of host immune cells have also been shown to be associated with the function of pyruvate kinase M2 (PKM2), a key metabolic regulator for glycolysis (102). Expression of PKM2, one of the two *Pkm/PKM* products, is upregulated during macrophage activation, while the cytosolic PKM1 that performs its catalytic function as a tetramer shows little change (102). In the cytoplasm, PKM2 exists primarily in an enzymatically inactive state by phosphorylation, and its dimer translocates into the nucleus, where it interacts with HIF-1α to activate target genes, including those encoding glycolytic enzymes and IL-1β (102). However, in activated macrophages, PKM2 activation by small molecules, such as TEPP-46, leads to the formation of a tetrameric structure that prevents its translocation into the nucleus, resulting in diminished Warburg effect and IL-1β production. This process also boosts the production of immune suppressive cytokine IL-10 and a decreased antimicrobial response, as observed against *S. typhimurinum* infection (102). In our studies, upregulation of *Pkm2/PKM2* in *Mtb*-infected murine macrophages, mouse and rabbit lungs as well as in the granulomas of human pulmonary TB patients (71) (Figures 1–3; Table 1) suggests that PKM2 has a similar regulatory role in facilitating the induction of HIF-1α-mediated Warburg effect and the associated antimicrobial response during *Mtb* infection. Finally, PHD3 has been shown to enhance glucose uptake and lactate production by facilitating the binding of PKM2 to HIF-1α, which activates HIF-1α-mediated gene expression (103).

In summary, it appears that the host immune cells mount a coordinated molecular signaling program to maximize the antimicrobial response during *Mtb* infection by integrating multiple mechanisms that lead to the induction of HIF-1α and the associated Warburg effect cascade. However, whether these mechanisms regulate cellular metabolism in different types/subtypes of immune cells during *Mtb* infection, how these regulatory mechanisms are integrated, and how they evolve during different stages of TB remain to be elucidated.

## Perturbing the Warburg Effect as a Mechanism of *Mtb* Pathogenicity?

### A Hypothesis

A switch in the bioenergetics from oxidative phosphorylation to the Warburg effect promotes M1 macrophage polarization that activates a Th1 adaptive response (16), both of which contribute to effective host defense against pathogens. However, the ability of *Mtb* to replicate and persist within the host cell suggests a novel survival mechanism, whereby the infecting *Mtb* perturbs the Warburg effect of host immune cells to dampen the M1 macrophage polarization. Similarly, interference of the metabolic switch in *Mtb*-infected DCs can also dampen their maturation and function. Modulation of the metabolic state in these antigen-presenting cells (APCs) by *Mtb* would thus diminish their ability to express MHC-II molecules and present antigens to CD4^+^ and CD8^+^ T cells, compromising activation, proliferation, and functions of the Th1 cellular response, which in turn would change M1/M2 polarization balance to favor the survival and persistence of the pathogen.

Multiple lines of evidence support our hypothesis presented above, including our observations from *in vitro* and *in vivo* studies of *Mtb* infection and from the lung granulomas of active TB patients. Alongside the expression of pro-inflammatory cytokine/chemokine and antimicrobial effector molecules, such as NOS2/iNOS, IL-12, and IL-1β, the concurrent induction of immune repressive IL-10 and ARG1 in *Mtb*-infected macrophages, lungs of mice and rabbits, and in the lung granulomas of active TB patients, suggests a suboptimal macrophage activation (24, 71, 77, 87, 104–106). Indeed, macrophage-derived IL-10 was shown to induce alternatively activated macrophages and promote exacerbation of chronic *Mtb* infection (105). A delayed Th1 immunity in pulmonary *Mtb* infection has been shown to be associated with the activation of immunoadaptor DAP-12-regulated IRAK-M and increased IL-10 expression in APCs (12). In addition, the differential expression of *ARG1* in lungs of rabbits infected by *Mtb* CDC 1551 and HN878 can be a contributing factor to the different infection outcome by the two strains. Indeed, *ARG1* was dampened early during *Mtb* CDC1551 infection of the rabbit lungs that ultimately develops latency, while *ARG1* expression was upregulated in the lungs of rabbits with active TB, which is associated with failed immune response (24, 25).

**Figure 4:**
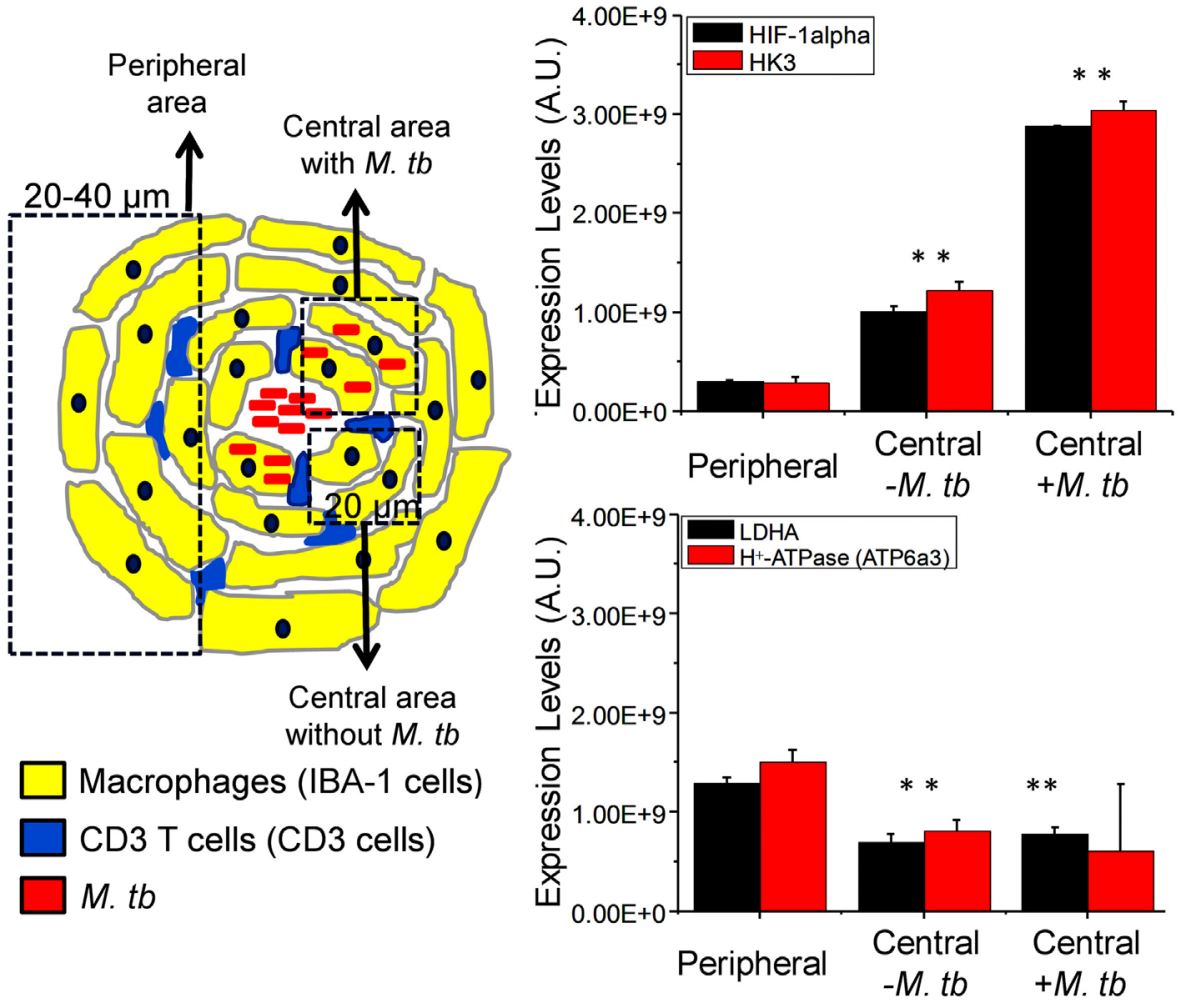
**Differential expression of HIF-1α, HK3, LDHA, and ATP6a3 of H^+^-ATPase in macrophages of infected mouse lung granulomas**. Immunohistochemical staining, confocal imaging, 3D reconstruction, and analysis were carried out using 20 μm lung tissue sections obtained from uninfected control and *Mtb*-infected C57BL/6 mice at day 30 postinfection. Tissue sections were stained for nuclei (DAPI), *Mtb*, target proteins, and macrophages (IBA-1). The expression of target protein was obtained by measuring the positive pixels and their intensities from equal number of cells of the specific regions of interests (ROIs) from the 3D reconstruction. Differential expression of target proteins relative to uninfected controls was carried out in macrophages at the center (within 20 μm in diameter) of granulomtous lesions with or without *Mtb* and in peripheral macrophages (20–40 μm in diameter). Three sections per lung region were examined for each animal and four animals were analyzed. Data shown are means ± SDs of the measurement from four mice. ** indicates *P* ≤ 0.005 between values obtained in macrophages at the center of granulomas in comparison to those at the peripheral areas. Data are from Shi et al. (71). Abbreviation: AU, arbitrary units.

More importantly, our hypothesis is further supported by the spatial differential expression of the Warburg effect-associated metabolic markers and HIF-1α in macrophages at different locations within mouse lung granulomas (Figure 4). Macrophages at the center of the granuloma, especially those infected with *Mtb*, showed significantly lower Warburg effect, as reflected by reduced expression of LDHA and H^+^-ATPase, compared to those in the periphery. In contrast, HIF-1α expression and glucose metabolism, as defined by increased expression of HK3, were higher in macrophages at the center of the granulomas than in those at the periphery (Figure 4). A reduced Warburg effect in the presence of high glucose metabolism in macrophages at the center of granulomas strongly suggests that *Mtb* perturbs the Warburg effect by siphoning off carbon flux, which should diminish the Warburg effect and hence result in suboptimal activation and functionality of infected macrophages. This notion is consistent with the findings that virulent *Mtb* strains induce more glucose uptake and perturb the glycolytic pathway of host cells, causing them to differentiate into foamy macrophage phenotype, compared to avirulent strains (107). Based on our observations, it is reasonable to suggest that therapeutic compounds that have the potential to enhance the Warburg effect in host immune cells, particularly in *Mtb*-infected macrophages, can be identified, developed, and used as anti-TB therapy.

In addition to TB, a deregulated immunometabolism has been found to be associated with pathogenicy of others pathogens, including HIV-1 (108–110). For example, in HIV-1-infected primary CD4^+^ T cells, a highly induced glycolytic capacity coupled with hyperreactive pro-inflammatory response was found to be essential for virion production (111, 112). Interestingly, in HIV-1-infected macrophages, the viral reservoirs, HIV-1 infection downregulates the Warburg effect by hijacking the metabolic function of HK1, thus conferring a survival advantage of host cell for the long-term viral persistence (113, 114).

### Roles of *Mtb* ESAT-6/CFP-10 in the Regulation of Host Immune Response and Cellular Metabolism

The different degree of virulence between *Mtb* CDC1551 and HN878 has been attributed to the presence of a phenolic glycolipid (PGL), a surface lipid of the complex *Mtb* cell wall that enhances virulence by increasing the infectivity of clinical *Mtb* isolates, especially in the W-Beijing strains (115). However, increasing evidence also suggests that *Mtb* ESAT-6/CFP-10 complex, one of the primary virulence factors of pathogenic strains, plays an important role in regulating host metabolism and immune response during *Mtb* infection. ESAT-6 and CFP-10 are two low molecular weight secreted proteins encoded by the region of difference 1 (RD1) of the *Mtb* genome (116). RD1 is absent in all vaccine strains of avirulent *M. bovis* BCG but is present in the virulent laboratory and clinical strains of *M. bovis* and *Mtb* (117). Findings from multiple studies also suggest that the ESAT-6/CFP-10 complex contributes to *Mtb* virulence by deactivating macrophage, dendritic, and T cell functions (118). Supporting evidence includes the requirement of ESAT-6/CFP-10 for *Mtb* replication and pathogenesis *in vivo* and the attenuated phenotype of *Mtb* mutants with no ESAT-6 production and/or secretion, including *Mtb Ra*, Δ*RD-1*, Δ*esat-6*, Δ*phoP*, and Δ*snm* (119–122).

Functionally, ESAT-6 has been shown to inhibit host cell TLR-signaling by directly binding to TLR2, resulting in reduced secretion of IL-12 p40 and TNF in macrophages (123). In addition, ESAT-6 was shown to be secreted into the cytosol of infected macrophages (124) and to induce the production of type I IFN (119, 125). Moreover, *Mtb* mutants, without *RD1* or *esat-6*, induce more robust pro-inflammatory cytokines in infected murine macrophages (119). In addition, exogenous ESAT-6 was found to induce host cell glucose uptake and perturbation of host cell glycolytic flux similar to that caused by virulent *Mtb* strains, leading to formation of the foamy macrophage phenotype in host cells (107, 126). These observations suggest that the role of ESAT-6/CFP-10 in dampening the pro-inflammatory and antimicrobial responses of immune cells is associated with their ability to interfere with the metabolic state of respective host cells. The interference of host metabolism by ESAT-6/CFP-10 is underscored by a recent report showing that ESAT-6/CFP-10 interacts directly with the host cell glycolytic enzymes ENO1 and PGK1, leading to the perturbation of glycolytic flux (126). It will be interesting to test whether the Warburg effect is enhanced in host cells infected by *Mtb* mutant strains with diminished ability to express and/or secrete ESAT-6 in comparison with wild-type and complemented strains.

## Conclusion

**Figure 5:**
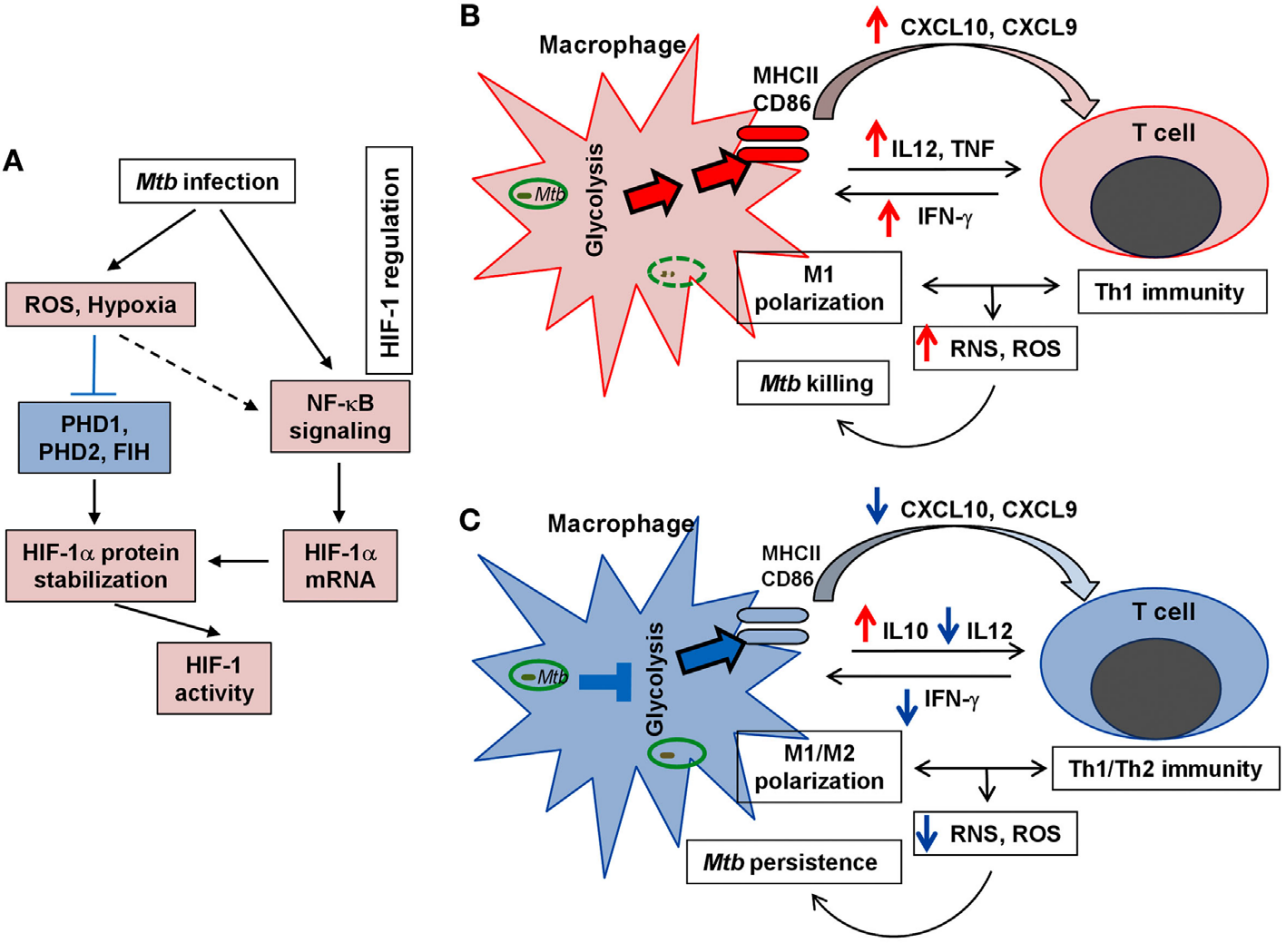
**Schematic representation of HIF-1 regulation and the Warburg effect in TB**. **(A)**. Key factors/steps leading to HIF-1 activation in response to *Mtb* infection. Blue block line indicates blockade and broken arrow shows indirect interaction. **(B)**. Upregulation of the Warburg effect state with effective M1 macrophage activation and efficient Th1 immunity, both of which lead to efficient *Mtb* clearance. **(C)**. Downregulation of the Warburg effect with compromised M1 macrophage polarization and dampened Th1 response that results in poor elimination of *Mtb* and promotes *Mtb* persistence. For **(A–C)**, red color (arrows and fill-in) indicates activation/upregulation and blue color denotes suppression/downregulation.

Activation of the Warburg effect in host immune cells in response to *Mtb* infection reveals a novel link between metabolic remodeling and host immune response, including the expression of antimicrobial and inflammatory immune responses. We postulate that the ability of pathogenic *Mtb* to compromise host bactericidal machinery by interfering with the metabolic switch to the Warburg effect can be a novel adaptation strategy for *Mtb* persistence and pathogenicity (Figure 5). Therefore, it is worth investigating the correlation between the macrophage polarization states, activation and differentiation of various T cell types/subtypes, and their bioenergetic pathways at different stages of *Mtb* infection. We anticipate that the evolving microenvironment within the granulomas regulates not only the immune response but also the local bioenergetic state. It is also important to define the correlation between the immune response and the Warburg effect in relation to the spatial localization of immune cells within different types of lung granulomas. A better understanding of immunometabolism in TB will provide promising avenue(s) for the development of novel therapeutic strategies that target the host cell metabolism to enhance antimicrobial and pro-inflammatory functions.

## Ethical Statement

### Mouse Study

All procedures involving live animals were performed in accordance with the Guide for Care and Use of Laboratory Animals of the National Institutes of Health, and individual procedures were approved by the Trudeau Institute Institutional Animal Care and Use Committee, as mentioned in Shi et al. (71).

### Rabbit Study

All experimental procedures with rabbits, including housing, infection with *Mtb*, postinfection care, necropsy, and processing of tissues were performed in BSL-3 facilities, as per the protocols approved by the Rutgers Biomedical and Health Sciences (RBHS; formerly UMDNJ) IACUC, as mentioned in Subbian et al. (24).

### Human Study

The protocols to recruit patients, collect, process, and analyze tissue were approved by the Health Sciences Ethics Committee of University of Cape Town, IRB of RBHS (formerly UMDNJ) and Cornell University, NY, as described in Subbian et al. (87).

## Author Contributions

LS and SS analyzed and interpreted the data from mouse lung, mouse bone marrow, rabbit, and human studies; EE performed, analyzed, and interpreted confocal imaging data. LS and SS wrote the manuscript. All authors read, edited, and agreed to publish the manuscript.

## Conflict of Interest Statement

The authors declare that the research was conducted in the absence of any commercial or financial relationships that could be construed as a potential conflict of interest.
